# Palm oil: a review on nutritional composition, processing, contaminants, and sustainability frameworks in the food system

**DOI:** 10.3389/fpls.2026.1798946

**Published:** 2026-05-05

**Authors:** Jerome Jeyakumar John Martin, Qi Wang, Mingming Hou, Xinyu Li, Xiaoyu Liu, Zhongming Fang, Chengxu Sun, Hongxing Cao

**Affiliations:** 1 National Key Laboratory for Tropical Crop Breeding, Chinese Academy of Tropical Agricultural Sciences, Haikou, China; 2 Coconut Research Institute, Chinese Academy of Tropical Agricultural Sciences, Wenchang, China; 3 Institute of Rice Industry Technology Research, Key Laboratory of Functional Agriculture of Guizhou Provincial Department of Education, Key Laboratory of Plant Resource Conservation and Germplasm Innovation in Mountainous Region (Ministry of Education), College of Agricultural Sciences, Guizhou University, Guiyang, China; 4 Horticulture Laboratory, School of Enology and Horticulture, Ningxia University, Yinchuan, Ningxia, China; 5 School of Life Sciences, Henan University, Kaifeng, Henan, China

**Keywords:** circular economy, food processing innovation, functional lipids, nutritional quality, palm oil, sustainable agriculture

## Abstract

Oil palm (*Elaeis guineensis* Jacq.) plays a vital role in the global food system, yet questions about its nutrition, safety, and sustainability continue to spark debate. This review takes a fresh, integrative approach, connecting what we know about nutrition, processing innovations, and sustainability governance within a single, systems-level framework. Palm oil is a rich source of energy and beneficial compounds like tocotrienols, carotenoids, and phytosterols, with research increasingly showing that its health impact depends on how it is consumed and processed. Advances in refining such as enzymatic degumming, low-temperature deodorization, and structured lipid design have significantly reduced harmful contaminants like 3-MCPD and glycidyl esters, sometimes by as much as 80%. On the sustainability side, challenges like deforestation, peatland conversion, and greenhouse gas emissions persist, even as certification programs (RSPO, MSPO, ISPO) and NDPE commitments make some progress. By weaving together these nutritional, technological, and environmental perspectives, this review offers a holistic roadmap for aligning palm oil production with global food security, climate action, and public health goals, providing actionable insights for researchers, policymakers, and industry leaders.

## 1 Introduction

Oil palm (*Elaeis guineensis* Jacq.) is vital to contemporary food systems due to its incomparable oil yield, multipurpose applications, and evolving value in alimentary and functional food science. Palm oil, derived from the fruit’s mesocarp and kernel, is a leading global commodity known not only for its economic and oxidative stability, but also for its widespread availability, which has been associated with increased per capita consumption of vegetable oils in many low- and middle-income nations (Food and Agriculture Organization of the United Nations (FAO), 2021). It has a high concentration of saturated and monounsaturated fatty acids, as well as lipid-soluble bioactives such as tocopherols, tocotrienols, carotenoids, and phytosterols, which have been studied for antioxidant, neuroprotective, and cholesterol-modulating effects (Alhaji et al., 2024). More recently, palm fatty acid distillate (PFAD) from refining by-products has been used as a concentrated source of vitamin E, adding emphasis to the worth of palm oil in nutraceutical applications (Pramana et al., 2024).

In terms of production, interspecific hybridization between *E. guineensis* and *E. oleifera* has resulted in cultivars with higher oleic acid content and heightened resistance to oxidative degradation, providing health and processing benefits (Afifi et al., 2025). Advances in genomics, enzyme-assisted processing, and lipid restructuring have enabled palm oil reformulations with specific melting profiles and lower contaminant loads (Weiss et al., 2025), thereby enabling product innovation aligned with health guidelines and consumer preferences.

Historically, oil palm cultivation originated in West Africa, with archaeological evidence indicating its use in the Niger Delta as early as 3000 BCE (Corley and Tinker, 2015). Industrial progress throughout the colonial era accelerated its spread to Southeast Asia, where Malaysia and Indonesia today account for more than 85% of global palm oil production (Food and Agriculture Organization of the United Nations (FAO), 2021). Emerging producers such as Thailand, Colombia, and sub-Saharan Africa are experiencing additional growth, owing to their agroecological appropriateness and mounting global demand.

The integration of palm oil into current food systems is powered by its affordability, physicochemical stability, and adaptability across a broad spectrum of edible and industrial products. It plays an integral role in the processed food industry, including margarine, bakery goods, frying oils, and non-dairy creamers, as well as in personal care, pharmaceuticals, and biofuels (Mancini et al., 2015). As of the 2024/25 marketing year, palm oil remains the most consumed vegetable oil worldwide, surpassing soybean, rapeseed, and sunflower oils in both production and demand. However, evolving trade policies and sustainability regulations, particularly in the EU, are reshaping global dynamics.

Despite its nutritional and economic value, the expansion of oil palm cultivation has sparked environmental and social debates. Land-use conversion, particularly on peatlands and in tropical forests, is associated with biodiversity loss and greenhouse gas emissions (Meijaard et al., 2020). Furthermore, working conditions on plantations remain under scrutiny. In response, certification schemes such as RSPO, MSPO, and ISPO aim to promote sustainable production standards, increase supply chain traceability, and protect labor rights, though implementation and verification remain challenging. Circular economy models, such as biogas from palm oil mill effluent (POME), biochar production from empty fruit bunches (EFB), and enzymatic valorization of palm kernel cake, are emerging as scalable mitigation solutions (Goh and Potter, 2022; Díaz-Orozco et al., 2025).

The central objective of this review is to critically examine the role of palm oil in contemporary food systems by addressing a key research question: to what extent can palm oil be responsibly integrated into modern food production while simultaneously balancing nutritional quality, technological functionality, and environmental sustainability? Despite its economic importance and functional advantages, palm oil remains at the center of ongoing scientific and policy debates concerning saturated fat intake, processing-induced contaminants, deforestation, and social governance challenges. This review synthesizes and critically evaluates current evidence on its compositional characteristics, health implications, processing innovations, and sustainability management strategies. By integrating these dimensions within a systems-level perspective, the study aims to provide a structured and evidence-based framework to inform researchers, policymakers, and industry stakeholders in developing balanced and sustainable pathways for palm oil utilization.

### 1.1 Methodology

**Figure 1:**
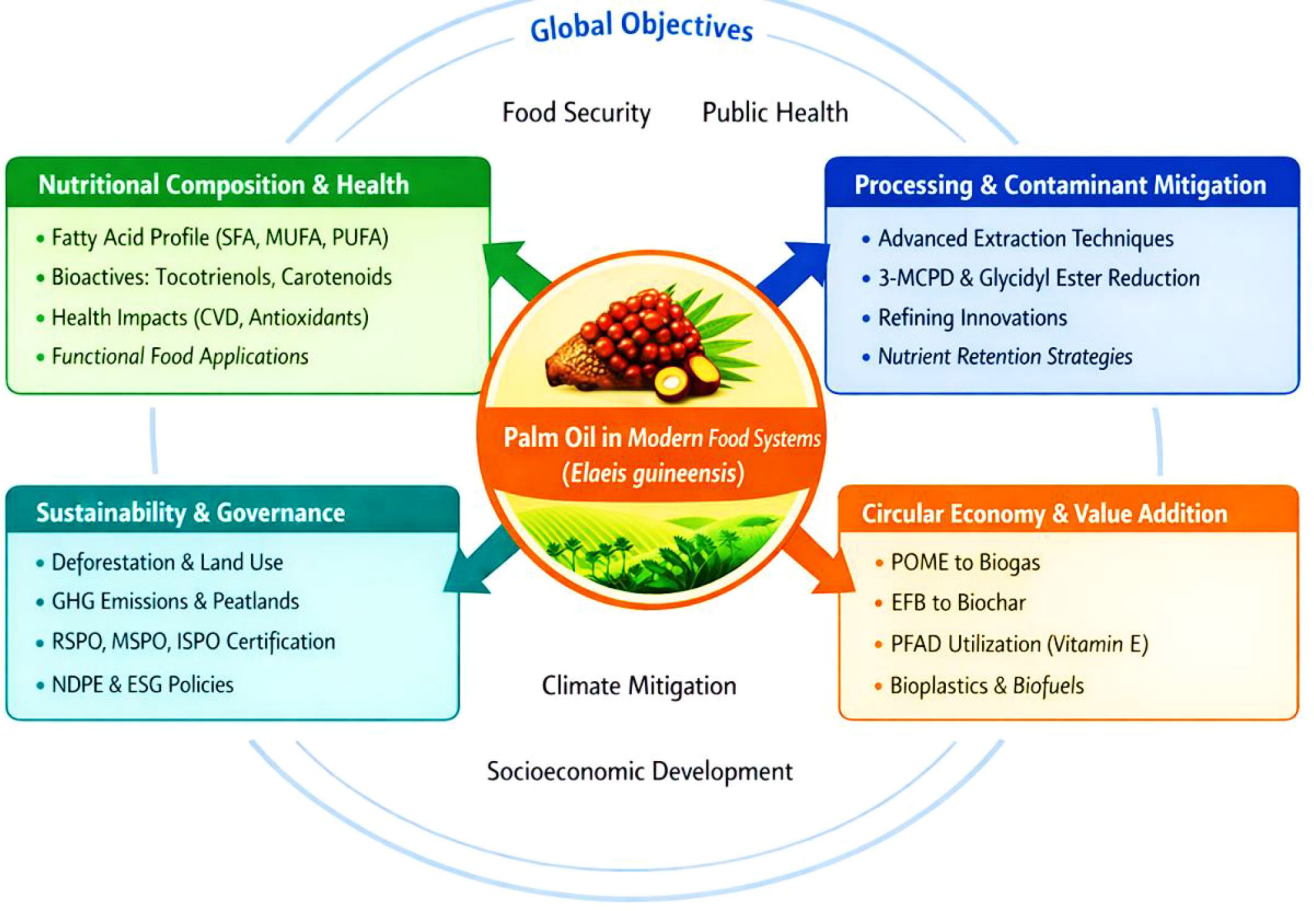
Holistic model aligning palm oil production with food security, public health, and environmental sustainability goals.

This study adopts a structured integrative review design to critically examine palm oil within the global food system through a sustainability-oriented and systems-based perspective (**Figure 1**). The review was conceived to synthesize interdisciplinary scholarship spanning nutritional biochemistry, food processing and safety engineering, environmental science, agricultural systems, and governance studies. Given the multidimensional nature of palm oil production and utilization, an integrative framework was selected to enable cross-sectoral analysis rather than a narrowly disciplinary assessment.

The analytical structure of the review was organized around three predefined and interrelated domains: (1) nutritional composition and health implications, (2) processing technologies and contaminant formation or mitigation, and (3) environmental impacts and sustainability governance mechanisms. These domains were selected because they represent the principal interfaces through which palm oil influences food system performance, including dietary quality, food safety, ecological integrity, economic viability, and institutional accountability.

Study selection was guided by criteria emphasizing scientific robustness, methodological clarity, and direct relevance to the defined domains. Priority was given to empirical investigations, mechanistic studies, life cycle assessments, risk evaluations, and policy analyses that contribute substantively to understanding structural trade-offs and systemic interactions. Conceptual and institutional analyses were included where they provided insight into governance frameworks, certification systems, or food system transitions.

The synthesis followed a two-stage analytical process. First, evidence was examined within each domain to identify prevailing findings, methodological trends, areas of scientific consensus, and points of contention. Second, cross-domain integration was undertaken to evaluate interactions among nutritional attributes, technological interventions, environmental externalities, and regulatory mechanisms. Particular attention was paid to identifying reinforcing feedbacks (e.g., technological innovation reducing environmental burdens), trade-offs (e.g., productivity versus land-use change), and policy leverage points capable of influencing system-wide outcomes.

This systems-based integrative approach enabled evaluation of palm oil not as an isolated commodity, but as a component embedded within broader food system dynamics. To enhance translational relevance and strategic clarity, the synthesized findings were subsequently consolidated through a structured SWOT framework in the concluding section. This framework facilitates a forward-looking assessment of internal sectoral characteristics and external sustainability drivers shaping palm oil’s future within resilient global food systems.

## 2 Major fatty acids in palm oil

The fatty acid (FA) and triacylglycerol (TAG) profile of palm oil renders it very suitable for different food applications (**Table 1**). Palm oil has a consistent combination of saturated and unsaturated fatty acids, with palmitic acid (C16:0) accounting for 44–47%, oleic acid (C18:1) for 36–40%, and linoleic acid (C18:2) for 9–12% (Prathap et al., 2025; Abd Alshafea et al., 2025). The product is structurally stable at high temperatures and semi-solid at ambient temperatures, making it effective for applications such as deep-frying and baking. Palm oil exhibits enhanced oxidative stability due to its higher proportion of saturated fatty acids. The potential of palmitic acid to increase LDL cholesterol levels has been a topic of health concern, particularly in Western dietary contexts. However, oleic acid, also abundant in palm oil, has been associated with improved HDL cholesterol levels in human observational studies, which may modulate the overall impact on lipid profiles when consumed in moderation (Ben Ayed et al., 2022). For instance, a comparative study noted that while palm oil is high in saturated fat, its substantial oleic acid content may balance its health impact within a varied diet (Xu et al., 2025). Controlled dietary intervention studies indicate that replacing palm oil with polyunsaturated fat–rich oils (such as sunflower or soybean oil) results in modest but consistent reductions in LDL cholesterol, typically in the range of 8–12%. At the same time, comparisons with animal-derived saturated fats (e.g., butter) demonstrate that palm oil often produces a more favorable lipid profile, including lower LDL concentrations and modest increases in HDL cholesterol. These findings position palm oil neither as categorically harmful nor neutral, but rather as metabolically intermediate among common dietary fats.

**Table 1:** Fatty acid composition (% by weight) of various palm oil types.

Fatty acid	Type	Common name	Approx. proportion (%)	Functional/nutritional role
Saturated				
Myristic acid (C14:0)	SFA	Myristic	~1	Minor component; contributes to texture and stability
Palmitic acid (C16:0)	SFA	Palmitic	44–47	Major determinant of oxidative stability; linked to LDL cholesterol increase
Stearic acid (C18:0)	SFA	Stearic	~5	Neutral effect on blood cholesterol; contributes to solid fat fraction
Unsaturated				
Oleic acid (C18:1)	MUFA	Oleic	36–40	Improves lipid metabolism and HDL levels; enhances shelf stability
Linoleic acid (C18:2)	PUFA	Linoleic	9–12	Essential fatty acid; maintains membrane fluidity
Linolenic acid (C18:3)	PUFA	α-Linolenic	<1	Omega-3 fatty acid; minor fraction but physiologically beneficial

### 2.1 Minor lipids and nutraceutical compounds in palm oil

Palm oil contain a wide spectrum of minor lipid constituents and bioactive nutraceuticals (**Table 2**) concentrated in the unsaponifiable fraction which accounts for nearly 1% of oil by weight, components have significant dietary, antioxidant, and beneficial implications. These compounds can be of two types glycerolipids such as monoglycerides, diglycerides, and phospholipid; and non-glycerolipids, carotenoids, phytosterols, squalene, and phenolic compounds, each of which contributes individually to palm oil’s biofunctionality (Alhaji et al., 2024; Teh and Lau, 2021). Red palm oil contains carotenoids, primarily α- and β-carotene, in quantities ranging from 500 to 1000 μg/g. These chemicals act as lipid-soluble antioxidants, inhibiting singlet oxygen and reducing oxidative stress in tissues. β-carotene from RPO is more bioavailable than synthetic vitamin A supplements, making RPO as an important dietary intervention for combating vitamin A deficiency in malnourished populations (Zainal et al., 2022). Additionally, Vitamin E in palm oil exists in two major classes: tocopherols and tocotrienols. While tocopherols are common in most vegetable oils, palm oil is unique in its abundance of tocotrienols, especially α-, γ-, and δ-tocotrienols, with concentrations reaching 500–800 µg/g in CPO. Tocotrienols demonstrate superior antioxidant potency compared to tocopherols and are involved in neuroprotection, cholesterol regulation, and anticancer activity through pathways such as HMG-CoA reductase inhibition and apoptosis induction (Ibrahim et al., 2025; Rodsamai et al., 2025). Studies using nanoencapsulation and liposomal carriers have shown improved stability and gastrointestinal absorption of these fragile molecules in functional foods.

**Table 2:** Bioactive compounds in palm oil.

Bioactive compound	Compound class	Health benefits	Mechanism of action	Natural source/origin in palm oil	References
α-,β-Carotene, Lycopene	Carotenoids (Terpenoids)	Antioxidant, supports vision, skin health, immune function	Scavenges singlet oxygen and peroxyl radicals; provitamin A activity	Red palm oil (RPO), palm olein	Tan et al. (2021); Abdullah et al. (2021)
Tocotrienols & Tocopherols	Vitamin E isoforms	Cardiovascular protection, anti-inflammatory, neuroprotection, anticancer	Inhibits lipid peroxidation, regulates gene expression, modulates lipid metabolism	Crude palm oil, PFAD, RPO	Zainal et al. (2022); Ismail et al. (2020)
β-sitosterol, Campesterol, Stigmasterol	Phytosterols (Sterols)	Lowers LDL cholesterol, immune modulation, anti-inflammatory	Competes with dietary cholesterol in intestinal absorption, influences cytokine pathways	PFAD, crude oil, deodorizer distillates	Makhmudova et al. (2021); Awad & Fink (2000)
Protocatechuic, Vanillic, p-Hydroxybenzoic Acid	Phenolic Acids	Antioxidant, anti-inflammatory, anticancer, neuroprotective	Inhibits NF-κB and COX pathways; scavenges radicals	Palm fiber oil, kernel cake, palm press fiber	Shahidi and Yeo (2018); Syarifah-Noratiqah (2019)
Catechins, Quercetin, Kaempferol	Flavonoids (Polyphenols)	Cardioprotective, anti-obesity, antidiabetic	Regulate endothelial NO production, modulate glucose transporters	Palm oil byproducts and residual fibers	Varatharajan et al. (2013); Manach et al. (2005)
Oleic, Linoleic, α-Linolenic Acids	Unsaturated Fatty Acids	Lipid-lowering, brain health, anti-inflammatory	Modulate membrane fluidity and prostaglandin synthesis	Palm olein, palm kernel oil	Schwab et al. (2021)
Squalene	Triterpene Hydrocarbon	Skin hydration, antioxidant, immune support	Prevents lipid peroxidation, protects cellular membranes	PFAD, red palm oil (minor)	Nurfatimah et al. (2021)

Phytosterols, such as β-sitosterol, campesterol, and stigmasterol, are present in amounts ranging from 200–300 mg/kg in crude palm oil. These compounds competitively inhibit cholesterol absorption in the intestine by mimicking endogenous sterol structures, effectively lowering serum LDL-cholesterol levels by 5–15% when incorporated into regular diets. Furthermore, their anti-inflammatory effects contribute to metabolic regulation, making them essential components in cardiovascular health-oriented functional food products (Teh and Lau, 2021).

Coenzyme Q10 (ubiquinone), although present in trace amounts (~10–20 µg/g), is vital for mitochondrial energy metabolism and shows promise in reducing oxidative injury in cardiac and muscular tissues. Squalene, a triterpenoid also found in palm oil, is a precursor to sterol biosynthesis and has been explored for skin barrier enhancement, anticancer properties, and use in vaccine adjuvants. Supercritical fluid extraction (SFE) techniques now allow for selective enrichment of these components from palm oil for high-value applications (Chañi-Paucar et al., 2025). Palm oil also contains oil palm phenolics (OPP),a water-soluble fraction derived during milling. This extract is rich in caffeoylshikimic acids, hydroxytyrosol, and other polyphenols. In clinical trials, OPP supplementation in individuals with mild hyperlipidemia significantly increased plasma total antioxidant capacity (TAC) and lowered pro-inflammatory cytokines such as IL-6 and TNF-α (Tadj et al., 2023). These nutraceutical compounds are increasingly being harnessed in functional food formulations, supplements, and clinical nutrition. Encapsulation of RPO in nanoliposomes and emulsions, as demonstrated by Rodsamai et al. (2025), ensures enhanced bioavailability, stability, and sensory neutrality, enabling the inclusion of bioactives in dairy products, beverages, and oral health supplements without compromising taste or shelf life.

Despite these benefits, the retention of minor components is highly sensitive to processing conditions. Conventional refining can reduce tocotrienol and carotenoid levels by up to 70%. Hence, cold-pressing, enzymatic degumming, and gentle deodorization are being promoted as industry best practices to preserve the nutritional value of palm oil-based products.

### 2.2 Role in functional foods and dietary applications

**Figure 2:**
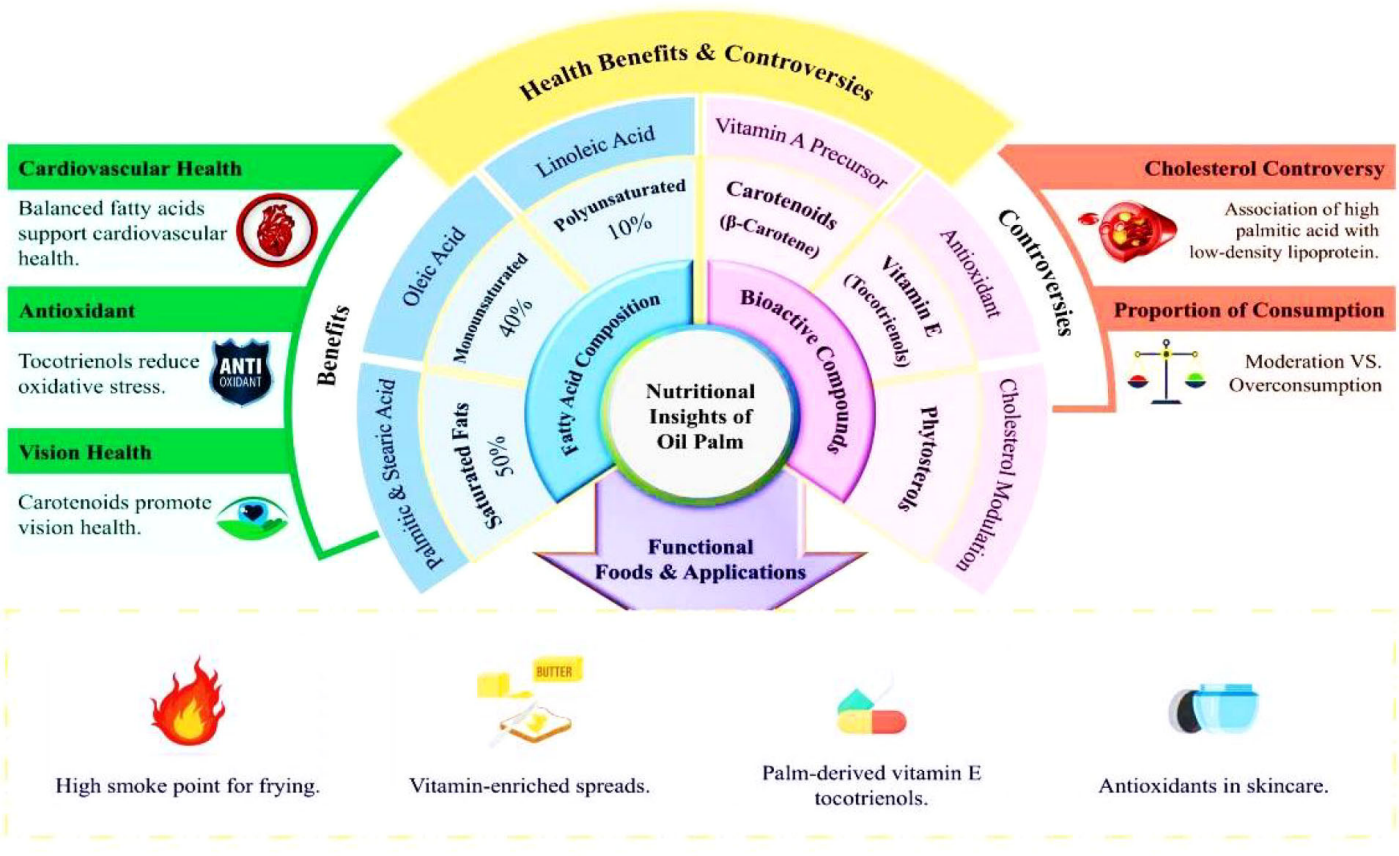
Comprehensive overview of the nutritional composition, health implications, and functional applications of oil palm-derived products.

Palm oil bioactives, including oil palm phenolics (OPP), are increasingly incorporated into functional foods due to their antioxidant and cholesterol-lowering properties (**Figure 2**). These compounds enhance health beyond basic nutrition, addressing conditions such as cardiovascular diseases (CVDs), neurodegenerative disorders, and immune dysfunction.

#### 2.2.1 Integration into fortified foods

The incorporation of palm oil and its bioactive fractions into fortified foods represents a strategic approach to enhance the nutritional quality of staple foods while addressing micronutrient deficiencies and chronic disease risk. A notable application is the fortification of margarine, cereal blends, and dairy products with red palm oil to combat vitamin A deficiency in regions such as Sub-Saharan Africa and South Asia. Clinical studies indicate that such fortification can improve serum retinol levels and reduce conditions like xerophthalmia and immune impairment (Choy et al., 2025).

To ensure the stability and bioavailability of lipid-soluble antioxidants during processing and storage, advanced delivery systems based on palm oil matrices have been developed. For instance, solid lipid microparticles (SLMs) formulated with palm stearin and stabilized by hydrolyzed soy protein isolate exhibit strong protective capacity, retaining a high proportion of bioactive compounds under extended storage at ambient and elevated temperatures (Brito-Oliveira et al., 2017). Similarly, spray-drying crude palm oil with gum arabic and cassava starch has been shown to preserve a substantial fraction of bioactives during prolonged storage at 45 °C, with even greater retention expected under standard conditions (Ferreira et al., 2016).

Beyond carotenoids and tocotrienols, other unsaponifiable constituents of palm oil known for their anti-inflammatory, neuroprotective, and lipid-regulating activities have been successfully incorporated into functional foods. Examples include nutraceutical bars, yogurt drinks, and lipid-based spreads targeted at populations with elevated risk of cardiovascular and neurodegenerative diseases. Innovations also extend to hybrid formulations that combine palm oil bioactives with other plant-based oils or natural emulsifiers, achieving synergistic antioxidant effects and improved functional properties. As consumer demand for clean-label and health-oriented products grows, palm oil’s diverse bioactive profile and compatibility with modern food-processing technologies offer a robust platform for developing next-generation functional foods across diverse markets.

#### 2.2.2 Palm oil derivatives in special diets

Palm oil derivatives have gained importance in specialized dietary regimens, including medical, ketogenic, and hypoallergenic diets, where tailored lipid profiles are essential to meet specific physiological and metabolic needs. A significant innovation is the production of medium-chain triglycerides (MCTs) from palm kernel oil (PKO). Unlike long-chain fatty acids, MCTs are rapidly absorbed and transported directly to the liver, where they are converted into ketone bodies—an important energy source for individuals with epilepsy, Alzheimer’s disease, or certain metabolic disorders.

A recent study by Manurung et al. (2025) demonstrated the enzymatic acidolysis of PKO to synthesize laurate-rich MCTs. Using immobilized lipase, optimal reaction conditions (94 °C for 24 h with a PKO-to-lauric acid molar ratio of 1:9) achieved 81.4% lauric acid incorporation, highlighting the potential of enzymatic processes to produce functional lipids for clinical and therapeutic nutrition.

Additionally, advanced analytical techniques, such as direct inlet negative ion chemical ionization tandem mass spectrometry, enable precise profiling of triacylglycerol regioisomers in palm oil. This allows identification of sn-2 palmitate-containing TAGs, which are crucial for improving fat digestibility and calcium absorption in infant formulas and clinical nutrition products, thereby enhancing nutrient bioavailability and reducing gastrointestinal discomfort in sensitive populations.

Palm oil also serves as a low-allergen lipid source in elimination diets, enteral feeds, and low-FODMAP regimens, owing to its freedom from common allergens (e.g., lactose, gluten, soy), neutral flavor, and high oxidative stability. Emerging evidence suggests that red palm oil derivatives may modulate gut microbiota and reduce inflammatory markers, indicating potential prebiotic and immunomodulatory effects (Bezerra et al., 2025). These attributes underscore the versatility of palm oil in specialized nutrition.

Despite these advantages, challenges related to consumer perceptions particularly concerning sustainability and saturated fat content persists. Transparent sourcing, credible sustainability certification, and clear nutritional labeling are essential to build consumer trust and foster wider adoption in clinical and specialty food sectors.

## 3 Advances in processing technologies

### 3.1 Innovations in palm oil extraction and refining technologies

**Figure 3:**
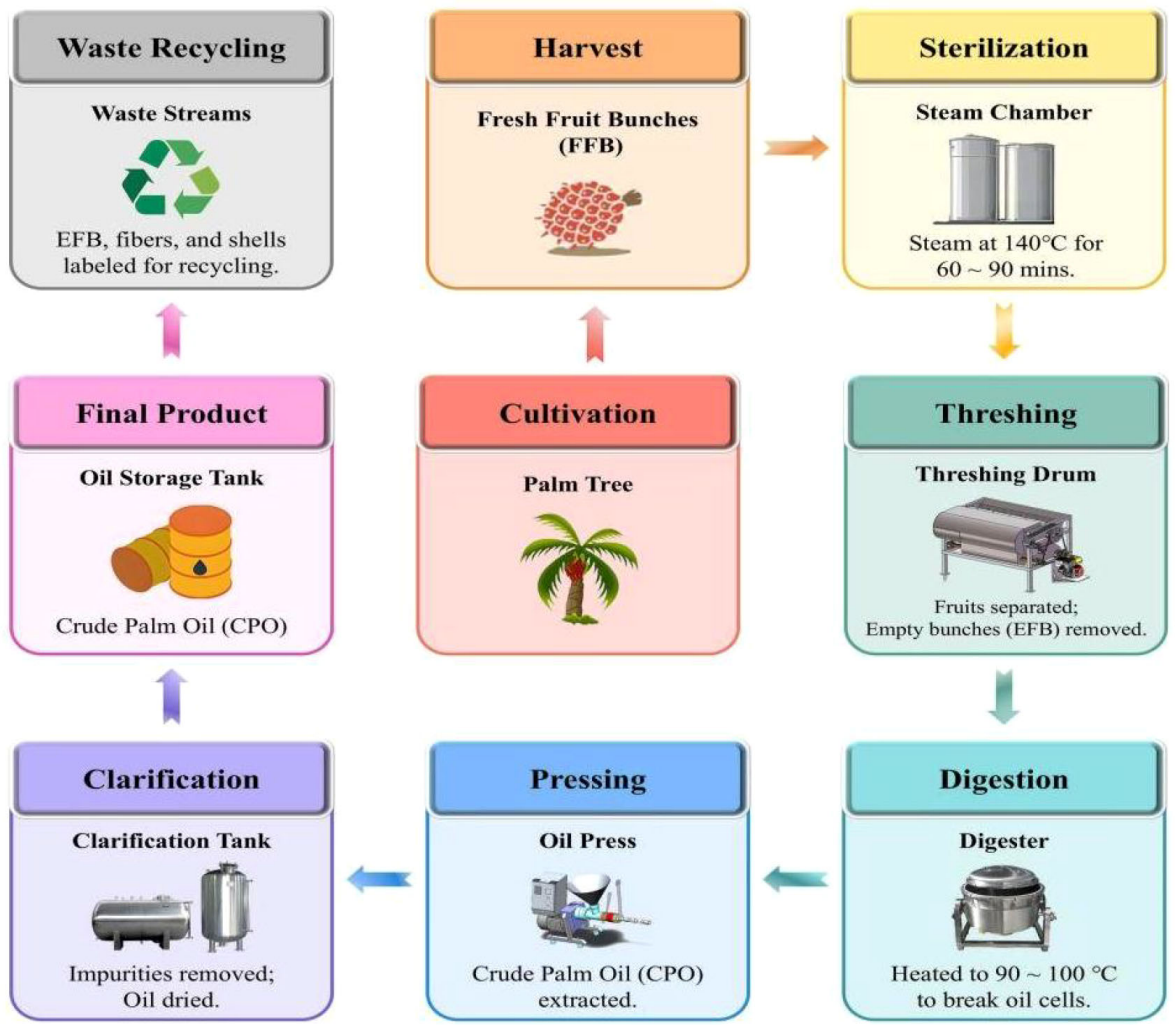
Schematic representation of palm oil handling and refinement workflow.

The palm oil industry has undergone significant technological transformation in the last decade, driven by the need for higher oil yields, bioactive preservation, environmental compliance, and circular economy integration. Traditional palm oil production, which involved batch-based thermal sterilization and mechanical pressing (**Figure 3**), is now being replaced or enhanced by digitally optimized, enzyme-assisted, and green extraction systems. These innovations span across harvest-to-refining chains, improving both processing efficiency and product quality (**Table 3**).

**Table 3:** Comparison of advanced palm oil extraction and refining technologies.

Technology	Core principle	Key advantages	Limitation	Applications	Reference
Enzyme-Assisted Extraction (EAE)	Hydrolytic enzymes (e.g., cellulase, pectinase) break down mesocarp cell walls	+ 10–12% oil yield, nutrient retention, lower temperature needed	Enzyme cost, reaction time, requires precise pH/temp control	Small/medium mills, nutraceutical-grade oil	Chew et al. (2021); Bakhshabadi et al. (2025)
Microwave-Assisted EAE	Microwave energy disrupts tissue, enhancing enzyme penetration	Fast heating, improved yield, reduces enzyme dosage	Requires optimization for uniform heating	Hybrid extraction, bioactive-rich oil	Abd Rashid et al., 2023
Supercritical CO_2_ Extraction (SFE)	Uses CO_2_ above critical point to extract bioactives without heat or solvents	Preserves carotenoids/tocotrienols, 95–98% purity, no solvent residues	High equipment cost, slower than conventional extraction	Cosmetic, pharmaceutical, functional food ingredients	Promraksa et al., 2020; Barbieri et al. (2025)
Enzymatic Degumming	Phospholipase A hydrolyzes phospholipids in crude oil	No chemical effluent, better for minor lipid retention	Slower than acid degumming	Physical refining for edible and bioactive oils	Nidzam et al., 2022
Short-Path Deodorization	Low-temp vacuum evaporation removes volatiles, preserves nutrients	Tocotrienol and carotenoid retention, reduced trans fats	Limited scale-up in some regions	Premium edible oils, minimally refined palm oil	Weiss et al. (2025)
Smart Sensor & AI-Based Optimization	NIR, FTIR, AI models adjust process parameters in real time	Precision control, yield maximization, process efficiency	High setup cost, requires data infrastructure	Modern industrial mills, export-quality processing	Mohamad Zaki et al. (2025); Akhtar et al. (2023)
Green Bleaching Earth Systems	Low-activation clay/carbon mixtures for adsorbing impurities	Lower oil loss, reduced contamination, regenerable material	May need more frequent replacement	Sustainable bleaching phase	Soetaredjo et al., 2021; Manurung et al. (2024)
Zero-Waste Biorefinery Concepts	Reuse of POME, EFB, PKS, soapstock, spent earth in secondary products	Reduces environmental load, generates electricity/fertilizer	Requires integration and capital investment	Full-chain sustainable palm oil refineries	Ahamed et al., 2023; Ali et al. (2015) Matseh 2018

#### 3.1.1 Mechanical extraction improvements

The earliest stage of oil processing mechanical extraction has been re-engineered to improve yield, reduce waste, and limit oxidative degradation. Modern systems now use fluidized bed sterilizers and continuous digester designs that allow better control of steam penetration and fruit softening. Grass Ramírez et al. (2023) reported that replacing traditional horizontal sterilizers with fluidized bed systems reduced thermal damage, leading to a 20% decrease in free fatty acid (FFA) formation and 15–20% increase in oil clarity. Upgraded screw press extractors—particularly the vertical twin-screw designs improve press cake dryness, enhancing recovery from mesocarp fibers. In advanced Malaysian mills, such upgrades have achieved extraction rates as high as 25%, compared to the national average of 20.5% (Chew et al., 2021). Additionally, vibration and cyclone-assisted de-oiling systems enable enhanced separation of oil from sludge and water phases, reducing product losses.

#### 3.1.2 Enzyme-assisted and low-energy extraction

Enzyme-assisted extraction (EAE) has emerged as a promising low-energy alternative to conventional mechanical and thermal palm oil extraction. This approach employs hydrolytic enzyme systems typically including pectinases, cellulases, and hemicellulases to partially degrade mesocarp cell wall polysaccharides, thereby facilitating oil release at reduced processing temperatures (approximately 45–55 °C). Compared with conventional hot-press extraction (>80 °C), EAE has been reported to improve oil extraction efficiency by ~8–12% on a fresh fruit bunch (FFB) mass basis, depending on enzyme formulation, dosage, and incubation time (Teixeira et al., 2013; Chew et al., 2021). Lower thermal exposure during EAE contributes to improved retention of thermolabile micronutrients, particularly carotenoids and tocotrienols, while limiting oxidative degradation. Several studies also indicate reductions in water and steam demand of approximately 15–20% relative to standard wet milling, highlighting the potential of EAE for decentralized or small-scale processing systems where energy and water availability are constrained. However, enzyme cost, reaction time, and downstream oil clarification remain key operational challenges, particularly for large-scale implementation. Recent developments integrate mild enzymatic pre-treatment with cold-pressing or aqueous extraction, producing crude palm oil fractions enriched in unsaponifiable components and suitable for nutraceutical or functional food applications. While these hybrid systems demonstrate improved bioactive retention, their overall mass yield and economic viability remain highly sensitive to enzyme recovery efficiency and process integration. Consequently, further optimization and life-cycle assessments are required to balance energy savings, oil quality, and scalability in commercial palm oil production.

#### 3.1.3 Refining innovations for nutrient retention

Refining is traditionally considered a nutrient-depleting phase in oil processing, with conventional chemical neutralization, bleaching, and high-temperature deodorization leading to losses of tocotrienols (up to 70%) and carotenoids. To counter this, green refining technologies have emerged. Enzymatic degumming is now widely studied and implemented. Using phospholipase A2 and B, this technique removes phospholipids without strong acids, reducing phosphorus content to below 10 ppm and preserving antioxidants (Yuslaini et al., 2023). Similarly, short-path molecular distillation and vacuum steam deodorization at 180–200 °C reduce degradation of minor lipids while removing off-flavors. Bleaching innovations involve the use of activated carbon-clay blends and regenerable bleaching earths that maintain oxidative stability and reduce process waste. These materials also enable partial adsorption of polyaromatic hydrocarbons and trace metals, enhancing oil purity for edible and cosmetic uses.

#### 3.1.4 Smart factory integration and real-time monitoring

The digital transformation of palm oil mills is accelerating through the adoption of Industry 4.0 technologies. Modern facilities increasingly incorporate inline sensors, near-infrared (NIR) spectrometers, and real-time oxidation index monitors to enable continuous process control. Akhtar et al. (2023) demonstrated that artificial intelligence (AI)-driven control systems can dynamically optimize key operational parameters—such as sterilization time, pressing torque, and refining temperatures—leading to reductions in energy consumption and labor costs while ensuring consistent product quality. Furthermore, these smart systems enhance traceability, a critical component of certified sustainable palm oil (CSPO) production. By digitally logging data at each processing stage, they strengthen supply chain transparency, improve accountability, and facilitate compliance with international sustainability standards such as RSPO (Roundtable on Sustainable Palm Oil) and ISCC (International Sustainability and Carbon Certification).

#### 3.1.5 Supercritical and green solvent extraction

Although not yet widely adopted in large-scale commodity oil production, supercritical CO_2_ extraction (SFE) is emerging as a promising technique in the nutraceutical and pharmaceutical industries. This method utilizes high-pressure carbon dioxide (~250–300 bar) at moderate temperatures (~40–60 °C) to selectively extract lipids, eliminating the need for hexane or excessive heat. Chañi-Paucar et al. (2025) demonstrated the efficacy of SFE in obtaining high-purity tocotrienols, carotenoids, and squalene—bioactive compounds of significant value in cosmetic, dietary supplement, and medical applications. While the high operational costs currently limit its feasibility for bulk oil production, SFE is particularly suitable for red palm oil fractionation aimed at isolating high-value bioactive components. As such, this technology aligns with industry efforts to diversify into functional oils and premium personal care ingredients, reinforcing the move toward value-added palm oil derivatives.

#### 3.1.6 Zero-waste and circular bioeconomy in processing

An emerging innovation in palm oil processing focuses on valorizing by-products to achieve zero-waste, circular bioeconomy models. This approach not only reduces environmental impact but also creates new revenue streams. For instance, empty fruit bunches (EFBs) are increasingly pelletized and combusted in biomass boilers to generate process heat and electricity, contributing to on-site energy self-sufficiency. Palm kernel shells (PKS) are utilized as feedstock in gasifiers or processed into high-value activated carbon for industrial applications. Sludge oil and soapstock by-products of refining are being converted into biodiesel or surfactants, supporting bioenergy and oleochemical sectors. Furthermore, spent bleaching earth, once considered hazardous waste, is now being reactivated through solvent washing for reuse, or repurposed in brick and cement manufacturing, reducing landfill burden and raw material consumption (Lai et al., 2024). Collectively, these strategies enhance the environmental sustainability of palm oil mills while aligning with global goals for waste reduction and resource circularity.

### 3.2 Contaminants in palm oil and their mitigation

Refining-induced contaminants, particularly 3-monochloropropane-1,2-diol esters (3-MCPDE) and glycidyl esters (GE), represent critical food safety concerns in palm oil due to their potential carcinogenicity and nephrotoxicity following gastrointestinal hydrolysis into free toxic compounds. These contaminants are primarily formed during high-temperature deodorization (≥240 °C) through reactions between acylglycerols, chloride precursors, and partial glycerides such as diacylglycerols (Sim et al., 2020; Yung et al., 2023). Recent industrial-scale mitigation strategies have focused on precursor removal, process optimization, and post-refining purification. For example, optimized refining conditions including controlled phosphoric acid degumming, reduced deodorization temperatures, and optimized bleaching earth dosage can reduce 3-MCPDE and GE levels by more than 65–80% without compromising oil quality (Sim et al., 2020; Yung et al., 2023).

Enzymatic degumming using phospholipases and lipases has emerged as a promising biotechnological approach by selectively hydrolyzing phospholipids and chlorine-containing precursors, thereby lowering contaminant formation while improving refining efficiency and oil –water separation (Liu et al., 2025). Adsorption-based technologies, including activated carbon, zeolites, and modified bleaching earth, have demonstrated contaminant removal efficiencies exceeding 90%, with some studies reporting up to 98% reduction depending on adsorbent composition and dosage (Yıldız et al., 2025). In addition, emerging green technologies such as enzymatic hydrolysis using immobilized lipases and deep eutectic solvent systems can remove approximately 80% of 3-MCPDE without adversely affecting oil quality, highlighting their potential for sustainable refining applications (Putra et al., 2023). Furthermore, advanced deodorization strategies, including low-temperature steam or short-path distillation, can reduce glycidyl esters by up to 99%, although optimization is required to maintain sensory and nutritional properties. Collectively, these recent advances demonstrate that integrated mitigation approaches combining precursor control, enzymatic processing, adsorption, and optimized refining conditions are essential to ensure palm oil safety while maintaining industrial scalability and nutritional quality.

### 3.3 Development of value-added products from palm oil and derivatives

**Figure 4:**
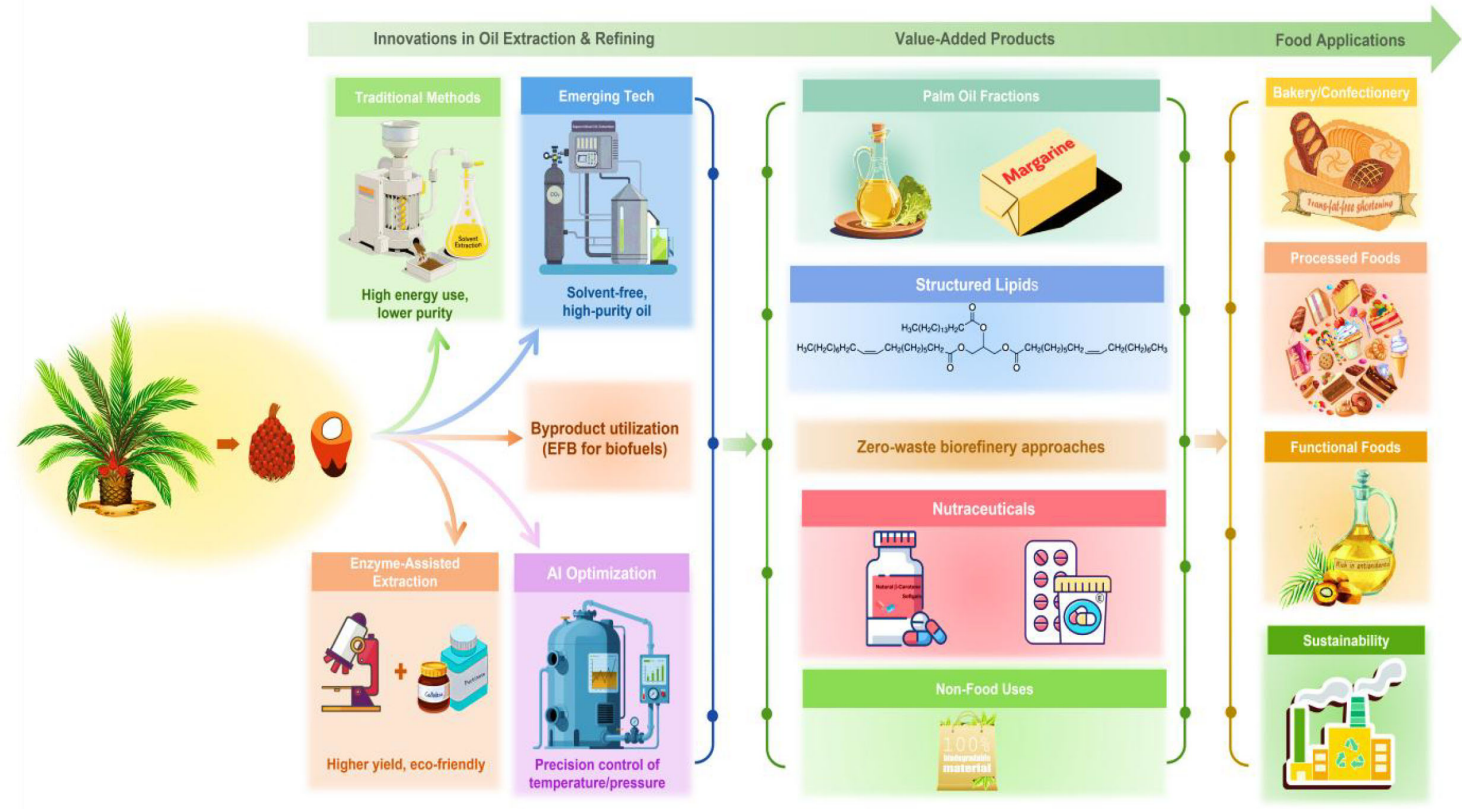
Innovations in palm oil processing and their role in value-added and sustainable applications.

The global palm oil industry is undergoing a strategic shift from bulk commodity production toward a value-addition model, aiming to optimize the use of oil and its by-products for higher economic, environmental, and health benefits. This transition is driven by rising global demand for sustainable bio-based products, increasing concerns about waste reduction, and the economic potential of palm-derived nutraceuticals, cosmeceuticals, biomaterials, and biofuels (**Figure 4**). Advanced bioprocessing and green chemistry innovations are central to this evolution, enabling palm oil derivatives to enter new sectors including pharmaceuticals, personal care, bioenergy, biodegradable plastics, and animal nutrition.

#### 3.3.1 Cosmeceuticals and biochemical ingredients

The cosmetic and pharmaceutical sectors increasingly exploit palm-derived glycerol, squalene, fatty esters, and coenzyme Q10 for skin hydration, anti-aging, and wound-healing formulations. Green extraction technologies such as supercritical CO_2_ and enzymatic esterification have enabled the production of high-purity emollients, emulsifiers, and lipophilic antioxidants suitable for dermatological use. Chuaitammakit et al. (2025) successfully synthesized hydroxymethylfurfural (HMF)-modified emulsifiers from crude palm oil, enhancing moisturization and product shelf life in cream-based formulations. Furthermore, palm-based tocotrienol-rich fractions (TRF) are now clinically tested for UV protection, melanin inhibition, and anti-inflammatory effects, supporting their inclusion in sun-care and anti-aging products.

#### 3.3.2 Biofuels and renewable energy products

Palm-derived materials such as PFAD, mesocarp sludge, and spent bleaching earth oil are now vital feedstocks for biofuels including biodiesel, green diesel, and bio-jet fuels. Through transesterification and hydrodeoxygenation (HDO), palm oil derivatives are converted into clean-burning renewable fuels. Puspitawati et al. (2025) noted that Malaysia and Indonesia’s B30 biodiesel mandates (30% palm oil blend) have stimulated national programs to develop downstream value-added biodiesel refineries, enhancing rural job creation and energy security while cutting GHG emissions by up to 60% compared to fossil diesel. Recent R&D also explores palm kernel shell gasification and biohydrogen production from palm waste, supporting the shift toward hydrogen economy integration in Southeast Asia.

#### 3.3.3 Green surfactants and household products

Oleochemical derivatives such as sodium lauryl sulfate, alkylpolyglucosides, and palm methyl ester sulfonates (MES) are derived from palm fatty acids and alcohols. These biodegradable compounds are replacing petroleum-based surfactants in eco-labelled household cleaning agents, including dishwashing liquids, laundry detergents, and shampoos. These palm-based alternatives exhibit superior foaming properties, lower toxicity, and are compatible with sensitive skin and aquatic ecosystems. Advancements in enzymatic esterification using lipases have improved the synthesis of mild palm-derived surfactants under low-energy, solvent-free conditions, making them suitable for “green chemistry” certified formulations.

#### 3.3.4 Bioplastics, resins, and polymers

Palm oil’s glycerol, stearic acid, and lignocellulosic waste serve as precursors for biodegradable plastics, thermoplastic starch blends, and bio-based polyols used in resins and foams. Beghetto (2025) emphasized the transformation of palm-derived glycerol into polyhydroxyalkanoates (PHAs) and poly(lactic acid) (PLA) blends, reducing reliance on fossil-based polymers. These materials are now used in packaging films, disposable utensils, and agricultural mulch films, offering biodegradability within 60–180 days under composting conditions. In construction, palm biomass-derived resins are incorporated into fiberboards, insulation panels, and eco-adhesives, supporting sustainable building materials.

#### 3.3.5 Animal feed and organic fertilizers

Palm kernel cake, empty fruit bunches (EFB), and mill effluent sludge are repurposed into fermented animal feed, ruminant silage, and bio-fertilizers. Through solid-state fermentation and enzymatic pre-treatment, these residues are enriched with protein and digestibility. Mairizal et al. (2025) showed that broiler chickens fed with fermented palm kernel meal displayed comparable growth performance to those fed with soybean meal, while reducing feed cost by 25–30%. EFB ash and palm boiler ash are also processed into soil conditioners rich in potassium, calcium, and trace elements, restoring soil fertility in degraded tropical lands.

## 4 Sustainability in oil palm production

### 4.1 Environmental and social challenges

The rapid expansion of oil palm plantations has both contributed significantly to global economies and resulted in serious ecological and social challenges. While palm oil remains the world’s most versatile and highest-yielding vegetable oil, its production has led to considerable environmental degradation and social conflicts, particularly in Southeast Asia. This section examines these challenges, comparing conventional oil palm production practices with emerging sustainable alternatives (**Table 4**).

**Table 4:** Conventional vs. sustainable palm oil production.

Aspect	Conventional palm oil production	Sustainable palm oil production
Land Use	Large-scale deforestation, often involving peatland drainage	Emphasis on no-deforestation policies and optimized land use (e.g., yield intensification)
Greenhouse Gas Emissions	High emissions due to forest clearing and peat oxidation	Reduced emissions via methane capture, no-burn practices, and carbon stock conservation
Biodiversity Impact	Significant habitat loss, threatening endangered species	Conservation of high conservation value (HCV) areas and wildlife corridors
Soil and Water Management	Soil erosion, nutrient depletion, and water contamination common	Implementation of soil conservation techniques and riparian buffer zones
Yield per Hectare	Variable; often low in aging plantations	Higher yields achieved through best agronomic practices and improved planting material
Waste Management	Often poorly managed, with palm oil mill effluent (POME) polluting water bodies	Waste valorization (e.g., POME biogas, composting, biofertilizers)
Pesticide and Fertilizer Use	Frequent use of chemical inputs, sometimes over-applied	Integrated pest management (IPM) and precision fertilization techniques
Labor Practices	Risk of exploitation, poor working conditions	Adherence to labor rights, fair wages, and worker safety standards
Traceability and Certification	Limited transparency and traceability	Certified schemes (e.g., RSPO, ISPO) with traceable and audited supply chains
Market Access	Vulnerable to regulatory restrictions and consumer backlash	Preferred by ethically conscious markets and multinational corporations

Sustainability in oil palm production faces significant environmental and social challenges, primarily arising from practices such as deforestation, habitat destruction, and pollution. The rapid expansion of oil palm cultivation, which has increased fourfold since the 1990s, has contributed to these ecological impacts, including habitat destruction, biodiversity loss, and the pollution of water systems from palm oil mill effluents (POME) (Meixner et al., 2023). Moreover, climate change and land resource scarcity complicate production further, impacting both the quantity and quality of palm oil (Kay Leng et al., 2024).

Social challenges, particularly labor rights issues, continue to affect the industry. Smallholders, often excluded from support mechanisms and fair compensation, face significant hardships, and land conflicts remain prevalent (Padfield et al., 2019). Addressing these challenges requires comprehensive sustainability strategies. Precision agriculture, sustainability certification programs, and enhanced stakeholder engagement are critical in mitigating negative impacts and promoting responsible production practices (Osei Darkwah and Ong-Abdullah, 2021).

### 4.2 Certification and sustainability initiatives in oil palm production

Sustainability certification initiatives are essential for mitigating the environmental and social challenges associated with oil palm cultivation. These certification programs establish guidelines to promote responsible practices across the supply chain, ensuring compliance with environmental, social, and economic sustainability criteria (**Table 5**). Among the most widely recognized certification schemes are the Roundtable on Sustainable Palm Oil (RSPO), the Malaysian Sustainable Palm Oil (MSPO), and the International Sustainability & Carbon Certification (ISCC). Each of these initiatives plays a distinct role in enhancing sustainability in palm oil production.

**Table 5:** Key legislation and certifications for sustainable oil palm cultivation.

Legislation/certification	Principles	Objectives	Coverage	Reference/website
RSPO	Environmental & social responsibility, legal compliance	Prevent deforestation, promote sustainability, human rights	Global	https://rspo.org
ISCC	GHG reduction, traceability, sustainable land use	Certify sustainable biomass, meet EU RED	Global	https://www.iscc-system.org
MSPO	Legal compliance, sustainability, continuous improvement	Align palm oil production with Malaysian & global standards	Malaysia	https://www.mspo.org.my
ISPO	Environmental/social responsibility, legality	Ensure legal, sustainable palm oil	Indonesia	https://www.bsigroup.com/en-ID/products-and-services/standards/ispo-indonesian-sustainable-palm-oil-system-certification/
EUDR	No deforestation, traceability, legality	Restrict import of deforestation-linked products	EU	https://eur-lex.europa.eu
US Lacey Act	No illegal timber/wildlife trade	Ensure legal sourcing of wood-based products	USA	https://www.fws.gov
Basel Criteria	Environmental protection, legality, transparency	Guide responsible palm oil sourcing	Global	https://wwf.panda.org/
HCS Approach	Forest conservation, carbon stock mapping	Protect high-carbon-value forests	Global	https://highcarbonstock.org
HCV Approach	Biodiversity, indigenous rights	Guide conservation-based land use	Global	https://hcvnetwork.org
Rainforest Alliance	Climate resilience, biodiversity, social equity	Promote sustainable agriculture	Global	https://www.rainforest-alliance.org
Fairtrade International	Ethical trade, smallholder empowerment	Ensure fair wages and community support	Global	https://www.fairtrade.net
NDPE Policies	No deforestation, peat, or exploitation	Guide ethical sourcing by corporations	Global	https://www.ndpe-irf.net/
POIG	Strengthen RSPO criteria	Drive leadership in palm oil sustainability	Global	https://poig.org
ASEAN Guidelines	Regional alignment, smallholder inclusion	Support ASEAN-wide sustainable palm oil	Southeast Asia	https://asean.org/book/asean-guidelines-on-sustainable-palm-oil/
APOI	Legal land rights, zero deforestation	Scale sustainable palm oil in Africa	Africa	https://www.tfa2020.org/en/african-palm-oil-initiative/
CESPO	Zero deforestation, social equity	Make Colombia a palm sustainability leader	Colombia	https://www.fedepalma.org
PEPPS	Biodiversity, community benefit	Promote low-impact palm oil in Peru	Peru	https://www.minam.gob.pe
IPEPS	Social inclusion, ecological resilience	Strengthen sustainability in Ecuador’s palm oil	Ecuador	https://www.ambiente.gob.ec/
TSPO	CSR, smallholder support, ecosystem protection	Transition to national sustainability standard	Thailand	https://www.dnp.go.th

The RSPO, established in 2004, is a multi-stakeholder initiative aimed at promoting the sustainable production and use of palm oil through a certification framework. RSPO certification is one of the most internationally recognized standards, encompassing principles related to environmental responsibility, social accountability, and economic viability. It ensures that palm oil is produced without contributing to deforestation, habitat destruction, or labor rights violations. Key principles include no deforestation, social responsibility, sustainable land use, and supply chain transparency. However, challenges such as enforcement and smallholder inclusion remain (Meijaard et al., 2020). The MSPO is a national certification standard developed by the Malaysian government to align palm oil production with global sustainability requirements. Unlike RSPO, MSPO certification is mandatory for all oil palm growers in Malaysia, ensuring nationwide adherence to sustainability principles. MSPO emphasizes environmental protection, regulatory compliance, social engagement, and continuous improvement. While it has strengthened Malaysia’s commitment to sustainable palm oil, challenges such as enforcement consistency and international recognition persist (Kay Leng et al., 2024). The ISCC is an internationally recognized sustainability certification scheme focused on carbon footprint reduction, supply chain transparency, and responsible land use. Widely used across multiple industries, including biofuels and food production, ISCC certification aligns with the EU Renewable Energy Directive (RED) and emphasizes greenhouse gas emissions reduction, supply chain traceability, ethical labor practices, and climate-smart agriculture (Alhaji et al., 2024). While beneficial for companies seeking compliance with stringent environmental regulations, the complexity and cost of certification pose challenges for smallholders and medium-scale producers (Meixner et al., 2023).

Despite the progress made through certification programs, several challenges persist in achieving fully sustainable palm oil production, including compliance enforcement, transparency, and market demand for certified palm oil. Additionally, smallholder farmers often struggle to meet certification requirements due to financial and technical constraints (Padfield et al., 2019). To enhance effectiveness, strategies such as strengthening enforcement mechanisms, increasing smallholder participation, advancing traceability technologies, and promoting consumer awareness are crucial. As global sustainability concerns intensify, certification schemes must continue evolving to ensure that palm oil production contributes positively to environmental conservation, social equity, and economic development.

### 4.3 Advancing sustainability in palm oil production through circular economy and digital innovations

The integration of circular economy (CE) principles into palm oil production has emerged as a pivotal strategy for enhancing sustainability by transforming waste materials into valuable resources, thereby reducing environmental impacts and increasing economic efficiency. Key by-products such as empty fruit bunches (EFB), palm oil mill effluent (POME), palm oil clinker (POC), and palm kernel shells are now repurposed through innovative applications that support both ecological and economic objectives. EFB, traditionally regarded as waste, is utilized for biomass energy generation and composting, reducing greenhouse gas emissions and enriching soil fertility (Bejarano et al., 2022), while POME undergoes anaerobic digestion to produce biogas, addressing both renewable energy demands and liquid waste management (Bejarano et al., 2022). Palm kernel shells are converted into biochar and activated carbon, contributing to soil improvement and water purification (Jagaba et al., 2021), whereas POC, a solid waste from incineration, finds versatile applications in green construction, asphalt production, wastewater treatment, and energy catalysis, thereby decreasing landfill use and promoting resource efficiency (Jagaba et al., 2021). The use of fertigation systems, where treated POME is recycled for irrigation, exemplifies nutrient recovery within plantation ecosystems, reducing dependence on synthetic fertilizers and lowering production costs. Cross-sectoral integration further enhances circularity, with optimization models highlighting synergies between the palm oil industry and sectors such as polymer and furniture manufacturing, leading to estimated economic gains exceeding USD 151 million and CO_2_ emission reductions of over 800,000 tons (Rajakal et al., 2023). However, the implementation of CE models faces multifaceted challenges, including high upfront investment costs, limited access to advanced processing technologies, insufficient digital infrastructure, workforce resistance to change, and fragmented regulatory frameworks (Siagian et al., 2024). In countries like Malaysia, CE progress is hindered by a lack of cohesive strategies and insufficient government incentives to support bio-waste valorization (Cheah et al., 2023). Addressing these issues requires collaborative efforts among policymakers, industry stakeholders, and research institutions, focusing on supportive legislation, technological infrastructure, and skill development. Digitalization plays a vital role in overcoming these barriers by enabling real-time monitoring, efficient waste management, and transparent supply chain operations through technologies like big data analytics, IoT, and AI. These tools enhance traceability, optimize resource flows, and facilitate cross-sector collaboration. Moreover, blockchain technology offers transformative potential by ensuring transparency, accountability, and secure data sharing across the supply chain. Smart contracts automate compliance with sustainability standards, while blockchain integration with geospatial and IoT data supports emissions monitoring and certification verification (El Hathat et al., 2023). This is particularly beneficial for empowering smallholders and promoting ethical sourcing through systems aligned with RSPO certification. Collectively, the adoption of CE principles, coupled with digital and blockchain innovations, signals a transition toward more resilient, inclusive, and environmentally sustainable palm oil production systems.

## 5 Challenges and opportunities

The palm oil industry, while economically significant, continues to face mounting health-related and environmental criticisms. These concerns stem primarily from industrial agricultural practices, land-use changes, and product health implications. Researchers and policymakers have increasingly focused on multidimensional strategies to mitigate these impacts through technological innovation, regulation, and sustainable landscape management. On the environmental front, palm oil production is closely associated with deforestation, biodiversity loss, greenhouse gas emissions, and water pollution. Expansion into peatlands remains one of the most damaging practices, releasing up to 120 tons of CO_2_ equivalents per hectare per year, exacerbating climate change. Additionally, the discharge of untreated palm oil mill effluent (POME) contaminates water bodies and contributes to eutrophication. Efforts to mitigate these effects include the implementation of methane capture systems, zero-burning policies, and buffer zone restoration, as highlighted by Goh and Potter (2022) and the IISD report (Voora et al., 2023). These strategies have been successful in some regions, cutting emissions by 50–70% and enhancing biodiversity in replanted buffer zones. Health-related concerns focus largely on the nutritional profile of palm oil and its links to cardiovascular risk when consumed excessively or in its refined form. While palm oil contains beneficial compounds such as tocotrienols and carotenoids, its high content of saturated fats has raised concerns. Regulatory bodies have responded by promoting moderate dietary use, developing low-saturation palm blends, and encouraging functional food formulations that integrate palm oil with healthier oils and bioactives. Toxicological concerns also arise from processing contaminants, such as 3-MCPD esters and glycidyl fatty acid esters, which form during high-temperature refining. These substances are classified as potentially carcinogenic. To address this, refining technologies are being upgraded to lower-temperature vacuum systems and enzymatic degumming methods, which reduce contaminant formation without compromising oil quality (Weiss et al., 2025). Social-environmental intersections also present challenges. Open burning and agrochemical misuse contribute to respiratory issues in nearby populations, especially in regions like Kalimantan and Riau. Strengthened enforcement of Good Agricultural Practices (GAPs), buffer planting zones, and community health monitoring are recommended and supported in the RSPO’s 2018 P&C revision. In terms of policy response, frameworks such as NDPE (No Deforestation, No Peat, No Exploitation) and national sustainability standards (e.g., MSPO, ISPO) have made progress in embedding health and environmental protections into certification schemes. However, as Voora et al. (2023) note, compliance and enforcement remain inconsistent, particularly among independent smallholders who face capacity and resource limitations. Bridging this gap will require not only financial and technical support but also inclusive policy design that accounts for smallholder constraints. The integration of circular economy models, including the conversion of palm residues into biogas, compost, and bioplastics, further reduces environmental pressure and opens health-conscious markets for clean-label and eco-certified palm-based products. When combined with digital innovations like IoT for chemical monitoring and blockchain for supply transparency, these strategies offer robust pathways to mitigate both health and ecological risks.

### 5.1 Strategic synthesis: SWOT analysis of palm oil in the global food system

To consolidate the multidisciplinary findings presented in this review, a SWOT (Strengths, Weaknesses, Opportunities, and Threats) framework was applied to evaluate palm oil production and utilization within a global food systems context. This strategic synthesis enables integration of nutritional, technological, environmental, and governance dimensions into a structured and forward-looking assessment.

### 5.2 Strengths

Palm oil demonstrates the highest oil yield per hectare among major vegetable oil crops, contributing significantly to land-use efficiency and global edible oil supply. Its physicochemical stability and semi-solid characteristics make it highly versatile for food processing applications, reducing the need for hydrogenation and associated trans-fat formation. Additionally, palm oil contains bioactive compounds such as tocotrienols, carotenoids, and phytosterols, which have been investigated for antioxidant and cardiometabolic properties. Its affordability and well-established global supply chains further reinforce its contribution to food security, particularly in low- and middle-income regions.

### 5.3 Weaknesses

Despite these advantages, palm oil remains subject to health-related debate due to its relatively high saturated fatty acid content. Refining processes may lead to the formation of contaminants such as 3-monochloropropane-1,2-diol (3-MCPD) and glycidyl esters, necessitating ongoing technological optimization. Environmentally, expansion of oil palm cultivation has been associated with deforestation, peatland conversion, biodiversity loss, and greenhouse gas emissions in certain producing regions. Governance gaps, uneven enforcement of certification standards, and traceability limitations further represent structural vulnerabilities.

### 5.4 Opportunities

Significant opportunities exist for sectoral transformation. Advances in plant breeding and biotechnology may enable development of high-oleic and climate-resilient varieties. Technological innovation in refining and contaminant mitigation can enhance food safety compliance and consumer confidence. Expansion and strengthening of sustainability certification systems, coupled with digital traceability tools, offer pathways for improved transparency and accountability. Integration of circular bioeconomy strategies such as valorization of palm oil mill effluent, empty fruit bunches, and refining by-products can enhance resource efficiency and reduce environmental burdens. Alignment with ESG frameworks and global sustainability targets provides additional momentum for reform.

### 5.5 Threats

External risks include increasingly stringent international regulations related to deforestation and carbon emissions, shifting consumer preferences toward perceived alternative oils, and geopolitical disruptions affecting global trade flows. Climate change poses additional uncertainty through altered rainfall patterns, pest pressures, and yield variability. Reputational risks and advocacy-driven campaigns may further influence market access and policy decisions.

## 6 Conclusion

Palm oil remains a pivotal component of the global agri-food system, valued for its functional properties, economic efficiency, and wide applicability. This review demonstrates that its role cannot be assessed through a single lens. Nutritionally, the impact of palm oil is context-dependent and influenced by overall dietary patterns and substitution effects rather than saturated fat content alone. Technological advancements in refining have significantly reduced contaminants such as 3-MCPD and glycidyl esters while improving bioactive retention, reflecting meaningful progress in processing safety.

At the same time, sustainability governance frameworks including certification systems and corporate commitments have contributed to improved traceability and deforestation risk mitigation, though implementation gaps and structural challenges persist.

Collectively, the findings highlight the need for an integrated, systems-level approach that aligns nutritional science, processing innovation, and environmental governance to ensure that palm oil production supports global food security, public health, and long-term environmental sustainability.

## References

[B1] Abd Rashid S. N. A. Leong H.-Y. Cheng K.-K. Yaakob H. Abdul Latiff N. (2023). Squalene-rich virgin palm oil by microwave-assisted enzyme aqueous extraction from palm mesocarp. Biocatal Agric. Biotechnol. 47, 102568. doi: 10.1016/j.bcab.2022.102568

[B2] Abdullah A. Atia A. Alrawaiq N. Kamil M. Yusof M. Rusli M. (2021). “ Palm oil tocotrienols in cancer chemoprevention and treatment,” in Vitamin E in health and disease (London: IntechOpen). doi: 10.5772/intechopen.98199

[B3] Afifi E. H. John Martin J. J. Wang Q. Li X. Liu X. Zhou L. . (2025). Fatty acid and lipid metabolism in oil palm: from biochemistry to molecular mechanisms. Int. J. Mol. Sci. 26, 2531. doi: 10.3390/ijms26062531 40141173 PMC11942028

[B4] Akhtar M. N. Ansari E. Alhady S. S. N. Abu Bakar E. (2023). Leveraging on advanced remote sensing- and artificial intelligence-based technologies to manage palm oil plantation for current global scenario: a review. Agriculture 13, 504. doi: 10.3390/agriculture13020504

[B5] Alhaji A. M. Almeida E. S. Carneiro C. R. da Silva C. A. S. Monteiro S. Coimbra J. S. R. (2024). Palm oil (Elaeis guineensis): a journey through sustainability, processing, and utilization. Foods 13, 2814. doi: 10.3390/foods13172814 39272579 PMC11394976

[B6] Alshafea M. Osman M. Galander A. Mekki M. (2025). Extraction and characterization of palm kernel oil from African oil palm (Elaeis guineensis) as a biodiesel feedstock in Sudan. Scholars Int. J. Chem. Mater. Sci. 8, 32–37. doi: 10.36348/sijcms.2025.v08i02.003

[B7] Awad A. B. Fink C. S. (2000). Phytosterols as anticancer dietary components: evidence and mechanism of action. J. Nutr. 130 (9), 2127–2130. doi: 10.1093/jn/130.9.2127 10958802

[B8] Bakhshabadi H. Ganje M. Gharekhani M. Mohammadi-Moghaddam T. Aulestia C. Morshedi A. (2025). A review of new methods for extracting oil from plants to enhance the efficiency and physicochemical properties of the extracted oils. Processes 13, 1124. doi: 10.3390/pr13041124

[B9] Barbieri M. B. Corrêa Junior D. Frases S. (2025). Fungal biotechnology applications in sustainable oil extraction. Appl. Microbiol. 5, 8. doi: 10.3390/applmicrobiol5010008

[B10] Beghetto V. (2025). Waste cooking oils into high-value products: Where is the industry going?. Polymers. 17 (7), 887. doi: 10.3390/polym17070887 40219276 PMC11991150

[B11] Bejarano P. A. C. Rodriguez-Miranda J. P. Maldonado-Astudillo R. I. Maldonado-Astudillo Y. I. Salazar R. (2022). Circular economy indicators for the assessment of waste and by-products from the palm oil sector. Processes 10, 903. doi: 10.3390/pr10050903

[B12] Ben Ayed R. Chirmade T. Hanana M. Khamassi K. Ercisli S. Choudhary R. . (2022). Comparative analysis and structural modeling of Elaeis oleifera FAD2, a fatty acid desaturase involved in unsaturated fatty acid composition of American oil palm. Biology 11, 529. doi: 10.3390/biology11040529 35453727 PMC9032008

[B13] Bezerra F. Salazar M. da Silva M. Souza S. Modesto E. Martins L. H. . (2025). Carotenoid-rich vegetable oils of palm trees from the Amazonia and their potential food applications. In Bioactive compounds (CRC Press), 191–223. doi: 10.1201/9781003462804-15

[B14] Brito-Oliveira T. C. Molina C. V. Netto F. M. Pinho S. C. (2017). Encapsulation of beta-carotene in lipid microparticles stabilized with hydrolyzed soy protein isolate: production parameters, alpha-tocopherol coencapsulation and stability under stress conditions. J. Food Sci. 82, 659–669. doi: 10.1111/1750-3841.13642 28182846

[B15] Chañi-Paucar L. O. Maceda Santivañez J. C. Paucarchuco Soto J. Portal-Cahuana L. A. Solis Malaga C. L. S. Chagua-Rodríguez P. . (2025). Supercritical fluid extraction of Amazonian oils and fats: promising species, equipment, yields, composition, and potential uses. Processes 13, 948. doi: 10.3390/pr13040948

[B16] Cheah W. Y. Siti-Dina R. P. Leng S. T. K. Er A. C. Show P. L. (2023). Circular bioeconomy in palm oil industry: current practices and future perspectives. Environ. Technol. Innovation 30, 103050. doi: 10.1016/j.eti.2023.103050

[B17] Chew C. L. Ng C. Y. Wai Onn H. Wu T. Lee Y. Y. Low L. E. . (2021). Improving sustainability of palm oil production by increasing oil extraction rate: a review. Food Bioproc Technol. 14, 1–19. doi: 10.1007/s11947-020-02555-1

[B18] Choy H. W. Teow S. J. Khor Y. P. Tan T. B. Mat Yusoff M. Gholivand S. . (2025). Optimisation and characterisation of red palm carotene-based microcapsules stabilised by rice protein isolate–flaxseed gum complex using various coating materials and core-to-wall ratios. Int. Food. Res. J. 32, 53–65. doi: 10.47836/ifrj.32.1.04

[B19] Chuaitammakit L. C. Poma W. Thepwatee S. Kaeopookum P. Samontha A. Thatumma N. . (2025). Value-added crude palm oil (CPO) functionalized with HMF for enhancing diesohol stability. Fuel 391, 134788. doi: 10.1016/j.fuel.2025.134788

[B20] Corley R. H. V. Tinker P. B. (2015). The oil palm. 5th Edn (Hoboken, NJ: Wiley-Blackwell). doi: 10.1002/9781118953297

[B21] Díaz-Orozco L. Moscosa Santillán M. Delgado Portales R. E. Rosales-Colunga L. M. Leyva-Porras C. Saavedra-Leos Z. (2025). Advances in L-lactic acid production from lignocellulose using genetically modified microbial systems. Polymers 17, 322. doi: 10.3390/polym17030322 39940524 PMC11820014

[B22] El Hathat Z. Venkatesh V. G. Sreedharan V. R. Tarik Z. Arunmozhi M. Shi Y. (2023). Analyzing the greenhouse gas emissions in the palm oil supply chain in a VUCA world: a blockchain initiative. Business Strat Environ 32 (8), 5563–5582. doi: 10.1002/bse.3436

[B23] Ferreira C. D. da Conceição E. J. L. MaChado B. A. S. Hermes V. S. de Oliveira Rios A. Druzian J. I. . (2016). Physicochemical characterization and oxidative stability of microencapsulated crude palm oil by spray drying. Food Bioproc Technol. 9, 124–136. doi: 10.1007/s11947-015-1603-z

[B24] Food and Agriculture Organization of the United Nations (FAO) (2021). The state of food and agriculture 2021: making agrifood systems more resilient to shocks and stresses (Rome: FAO). doi: 10.4060/cb4476en

[B25] Goh C. S. Potter L. (2022). Bio-economy for sustainable growth in developing countries: the case of oil palm in Malaysia and Indonesia. Biofuel Bioprod Biorefin 16, 1808–1819. doi: 10.1002/bbb.2381

[B26] Grass Ramírez J. F. Muñoz R. C. Zartha Sossa J. W. (2023). Innovations and trends in the coconut agroindustry supply chain: a technological surveillance and foresight analysis. Front. Sustain. Food. Syst. 7. doi: 10.3389/fsufs.2023.1048450

[B27] Ibrahim N.‘. Muhammad Ismail Tadj N. B. Leow S. S. Nagalingam T. Tg Abu Bakar Sidik T. M. I. Haji Mohd Saad Q. . (2025). Oil palm phenolics supplementation improves plasma antioxidant levels and modulates inflammatory markers in individuals with minor hyperlipidaemia: a randomized, double-blind, placebo-controlled trial. J. Funct. Food 128, 106810. doi: 10.1016/j.jff.2025.106810

[B28] Ismail M. Alsalahi A. Imam M. U. Ooi D. J. Khaza'ai H. Aljaberi M. A. . (2020). Safety and Neuroprotective Efficacy of Palm Oil and Tocotrienol-Rich Fraction from Palm Oil: A Systematic Review. Nutrients 12 (2), 521. doi: 10.3390/nu12020521 32085610 PMC7071496

[B29] Jagaba A. H. Kutty S. R. M. Hayder A. P. T. D. G. Noor A. Hafiz M. Aliyu Yaro N. S. . (2021). “ Palm oil clinker as a waste by-product: utilization and circular economy potential,” in IntechOpen ( IntechOpen, London), 1–24. doi: 10.5772/intechopen.97312

[B30] Kay Leng S. T. Yan C. Pahri S. Lin N. Choy E. R. (2024). A systematic literature review on environmental issues and challenges towards the palm oil industry. J. Sustainab Sci. Manage. 19, 154–170. doi: 10.46754/jssm.2024.01.013

[B31] Lai V. Yusoff N. Y. M. Ahmed A. N. Huang Y. F. Boo K. B. W. El-Shafie A. (2024). The benefits and perspectives of the palm oil industry in Malaysia. Environ. Dev. Sustain. 27, 15235–15249. doi: 10.1007/s10668-024-04593-7

[B32] Liu X. Huang C. Lan D. Wang W. Wang Y. (2025). Combining phospholipase A1 with monoacylglycerol lipase for crude vegetable oil degumming through improved oil-water separation. Food Chem. 467, 142366. doi: 10.1016/j.foodchem.2024.142366 39644657

[B33] Mairizal A. Akmal Fahmida Filawati Ananda T. G. (2025). Growth performance and chemical quality of broiler meat fed with fermented palm kernel meal based ration supplemented with methionine and lysine. Journal of Advanced Veterinary and Animal Research 12 (1), 1–8. 40568517

[B34] Makhmudova U. Schulze P. C. Lütjohann D. Weingärtner O. (2021). Phytosterols and Cardiovascular Disease. Current Atherosclerosis Reports 23. doi: 10.1007/s11883-021-00964-x PMC8410723 34468867

[B35] Manach C. Williamson G. Morand C. Scalbert A. Rémésy C. (2005). Bioavailability and bioefficacy of polyphenols in humans. I. Review of 97 bioavailability studies. The American journal of clinical nutrition 81 (1 Suppl), 230S–242S. doi: 10.1093/ajcn/81.1.230S 15640486

[B36] Mancini A. Imperlini E. Nigro E. Montagnese C. Daniele A. Orrù S. . (2015). Biological and nutritional properties of palm oil and palmitic acid: effects on health. Molecules 20, 17339–17361. doi: 10.3390/molecules200917339 26393565 PMC6331788

[B37] Manurung A. I. Julianti E. Silalahi J. Siahaan D. (2025). Synthesis of lauric-rich medium-chain triglycerides from palm kernel oil and lauric acid by enzymatic acidolysis. J. Oil Palm Res. 37, 1–10. doi: 10.21894/jopr.2025.0010

[B38] Manurung R. Maisarah S. Harahap H. Parinduri S. Pranata A. (2024). Reactivated spent bleaching earth as a new path in waste resource utilization for the crude palm oil bleaching process. E3S Web of Conferences 560, 01005. doi: 10.1051/e3sconf/202456001005

[B39] Meijaard E. Brooks T. M. Carlson K. M. Slade E. M. Garcia-Ulloa J. Gaveau D. L. A. . (2020). The environmental impacts of palm oil in context. Nat. Plants 6, 1418–1426. doi: 10.1038/s41477-020-00813-w 33299148

[B40] Meixner O. Hackl S. Haas R. (2023). Assessing the sustainability of palm oil by expert interviews—an application of the analytic hierarchy process. Sustainability 15, 16954. doi: 10.3390/su152416954

[B41] Mohamad Zaki M. A. Ooi J. Ng W. P. Q. How B. S. Lam H. L. Foo D. C. Y. . (2025). Impact of industry 4.0 technologies on the oil palm industry: a literature review. Smart Agric. Technol. 10, 100685. doi: 10.1016/j.atech.2024.100685

[B42] Nidzam M. S. Hossain M. S. Ismail N. Abdul Latip R. Mohammad Ilias M. K. Mobin Siddique M. B. . (2022). Influence of the degumming process parameters on the formation of glyceryl esters and 3-MCPDE in refined palm oil: optimization and palm oil quality analyses. Foods 11, 124. doi: 10.3390/foods11010124 35010250 PMC8750379

[B43] Nurfatimah R. P. Ahmadi K. G. S. Hapsari I. Kholila K. Estiasih T. (2021). Separation of squalene-rich fraction from palm oil fatty acid distillate (PFAD): a review. IOP Conf. Ser. Earth Environ. Sci. 733, 12094. doi: 10.1088/1755-1315/733/1/012094

[B44] Osei Darkwah D. Ong-Abdullah M. (2021). “ Sustainability of the oil palm industry,” in Elaeis guineensis. Ed. Kamyab H. ( IntechOpen, London). doi: 10.5772/intechopen.100156

[B45] Padfield R. Hansen S. Davies Z. G. Ehrensperger A. Slade E. M. Evers S. . (2019). Co-producing a research agenda for sustainable palm oil. Front. For. Global Change 2. doi: 10.3389/ffgc.2019.00013

[B46] Pramana A. Kurnia D. Firmanda A. Rossi E. Ar N. H. Putri V. J. (2024). Using palm oil residue for food nutrition and quality: from palm fatty acid distillate to vitamin E toward sustainability. J. Sci. Food Agric. 105 (9), 4728–4740. doi: 10.1002/jsfa.13878 39258508

[B47] Prathap V. Yadav P. Manorama K. Ravichandran G. Suresh K. Mathur R. (2025). Optimizing fatty acid composition and nutritional profile of palm oil-based blends for improved functional properties. J. Food Meas Charact 19, 2861–2878. doi: 10.1007/s11694-025-03152-6

[B48] Promraksa A. Siripatana C. Rakmak N. Chusri N. (2020). Modeling of supercritical CO_2_ extraction of palm oil and tocopherols based on volumetric axial dispersion. J. Supercrit Fluids 166, 105021. doi: 10.1016/j.supflu.2020.105021

[B49] Puspitawati E. Nurdianto N. R. Pambudi A. Alamsyah M. R. Pakerti K. A. Maharani N. D. . (2025). Economic effect of biodiesel downstream industry: An analysis based on a dynamic CGE model. International Journal of Energy Economics and Policy 15 (1), 437–446. doi: 10.32479/ijeep.17428

[B50] Putra S. S. S. Basirun W. J. Hayyan A. Elgharbawy A. A. M. (2023). Enzymatic hydrolysis for the removal of 3- monochloropropanediol esters in edible oils using Candida rugosa lipase in the presence of deep eutectic solvents and nanocellulose. Biochem. Eng J. 196, 108958. doi: 10.1016/j.bej.2023.108958

[B51] Rajakal J. Hwang J. Hassim M. Andiappan V. Tan Q. Ng D. K. S. (2023). Integration and optimisation of palm oil sector with multiple industries to achieve circular economy. Sustain. Prod. Consumption 40, 1–14. doi: 10.1016/j.spc.2023.06.022

[B52] Rodsamai T. Chaijan M. Rodjan P. Tamman A. Supaweera N. Yin M. . (2025). Design and bioanalysis of nanoliposome loaded with premium red palm oil for improved nutritional delivery and stability. Foods 14, 566. doi: 10.3390/foods14040566 40002010 PMC11854538

[B53] Schwab U. Reynolds A. Sallinen T. Rivellese A. Risérus U. (2021). Dietary fat intakes and cardiovascular disease risk in adults with type 2 diabetes: a systematic review and meta-analysis. Eur. J. Nutr. 60 (6), 3355–3363. doi: 10.1007/s00394-021-02507-1 33611616

[B54] Shahidi F. Yeo J. (2018). Bioactivities of phenolics by focusing on suppression of chronic diseases: a review. Int. J. Mol. Sci. 19, 1573. doi: 10.3390/ijms19061573 29799460 PMC6032343

[B55] Siagian U. Wenten I. G. Khoiruddin K. (2024). Circular economy approaches in the palm oil industry: enhancing profitability through waste reduction and product diversification. J. Eng. Technol. Sci. 56, 25–49. doi: 10.5614/j.eng.technol.sci.2024.56.1.3

[B56] Sim B. I. Khor Y. P. Lai O. M. Yeoh C. B. Wang Y. Liu Y. . (2020). Mitigation of 3-MCPD esters and glycidyl esters during the physical refining process of palm oil by micro and macro laboratory scale refining. Food Chem. 328, 127147. doi: 10.1016/j.foodchem.2020.127147 32497897

[B57] Soetaredjo F. E. Laysandra L. Putro J. N. Santoso S. P. Angkawijaya A. E. Yuliana M. . (2021). Ecological-safe and low-cost activated-bleaching earth: Preparation, characteristics, bleaching performance, and scale-up production. Journal of Cleaner Production 279, 123793. doi: 10.1016/j.jclepro.2020.123793

[B58] Syarifah-Noratiqah S. B. Zulfarina M. S. Ahmad S. U. Fairus S. Naina Mohamed I. (2019). The pharmacological potential of oil palm phenolics (OPP) individual components. International Journal of Medical Sciences 16, 711–719. doi: 10.7150/ijms.29934 31217739 PMC6566743

[B59] Tadj MI N. B. Ibrahim N. I. Tg Abu Bakar Sidik T. M. I. Zulfarina M. S. Haji Mohd Saad Q. Leow S.-S. (2023). Safety and efficacy of oil palm phenolic supplementation in improving lipid profile among hyperlipidemic adults: A phase 2, randomized, double-blind, placebo-controlled clinical trial. Frontiers in Pharmacology 14. doi: 10.3389/fphar.2023.1190663 PMC10360129 37484009

[B60] Tan C. H. Lee C. J. Tan S. N. Poon D. T. S. Chong C. Y. E. Pui L. P. (2021). Red palm oil: a review on processing, health benefits and its application in food. J. Oleo Sci. 70, 1201–1210. doi: 10.5650/jos.ess21108 34373407

[B61] Teh S. S. Lau H. L. N. (2021). Quality assessment of refined red palm-pressed mesocarp olein. Food Chem. 340, 127912. doi: 10.1016/j.foodchem.2020.127912 32916404

[B62] Teixeira C. B. Macedo G. A. Macedo J. A. Da Silva L. H. da C. Rodrigues A. M. (2013). Simultaneous extraction of oil and antioxidant compounds from oil palm fruit (Elaeis guineensis) by an aqueous enzymatic process. Bioresour Technol. 129, 575–581. doi: 10.1016/j.biortech.2012.11.057 23274221

[B63] Varatharajan R. Sattar M. Z.A. Chung I. Abdulla M. A. Kassim N. M. Abdullah N. A. (2013). Antioxidant and pro-oxidant effects of oil palm (Elaeis guineensis) leaves extract in experimental diabetic nephropathy: A duration-dependent outcome. BMC Complementary and Alternative Medicine 13, 242. doi: 10.1186/1472-6882-13-242 24074026 PMC3829664

[B64] Voora V. Bermudez S. Farrell J. Larrea C. Luna E. (2023). “Palm oil prices and sustainability,” in Palm oil prices and sustainability (International Institute for Sustainable Development, Winnipeg).

[B65] Weiss J. Mannweiler S. Salminen H. (2025). Precision processing for value-added fats and oils. Annu. Rev. Food Sci. Technol. 16 (1), 39–61. doi: 10.1146/annurev-food-111523-121237 39899844

[B66] Xu W. John Martin J. J. Li X. Liu X. Cheng S. Cao H. (2025). Transcriptional and metabolic analysis of oleic acid synthesis in seedless and tenera oil palm species. Front. Plant Sci. 16. doi: 10.3389/fpls.2025.1557544 PMC11893603 40070716

[B67] Yıldız K. Özdikicierler O. Günç Ergönül P. (2025). Investigating the role of adsorbent type and ratio in mitigating 3-MCPD and GE formation during the inhibition of palm oil chemical interesterification via earth treatment. Food Chem. 476, 143395. doi: 10.1016/j.foodchem.2025.143395 39987801

[B68] Yung Y. L. Lakshmanan S. Kumaresan S. Chu C. M. Tham H. J. (2023). Mitigation of 3-monochloropropane 1,2 diol ester and glycidyl ester in refined oil – A review. Food Chem. 429, 136913. doi: 10.1016/j.foodchem.2023.136913 37506659

[B69] Yuslaini N. Sumadinata R. Fedryansyah M. Abdillah A. Prianto A. L. Febriyanti D. (2023). Sustainable investment strategies in the palm oil industry in Indonesia. J. Infrastruct Policy Dev. 7, 2288. doi: 10.24294/jipd.v7i3.2288

[B70] Zainal Z. Khaza’ai H. Kutty Radhakrishnan A. Chang S. K. (2022). Therapeutic potential of palm oil vitamin E-derived tocotrienols in inflammation and chronic diseases: evidence from preclinical and clinical studies. Food Res. Int. 156, 111175. doi: 10.1016/j.foodres.2022.111175 35651097

